# Draft Genome Sequence of the Carboxydotrophic Alphaproteobacterium Aminobacter carboxidus Type Strain DSM 1086

**DOI:** 10.1128/MRA.01170-20

**Published:** 2020-11-05

**Authors:** Paolo Turrini, Irene Artuso, Marco Tescari, Gabriele Andrea Lugli, Emanuela Frangipani, Marco Ventura, Paolo Visca

**Affiliations:** a Department of Science, Roma Tre University, Rome, Italy; b Laboratory of Probiogenomics, Department of Chemistry, Life Sciences, and Environmental Sustainability, University of Parma, Parma, Italy; c Department of Biomolecular Sciences, University of Urbino Carlo Bo, Urbino, Italy; Portland State University

## Abstract

Aminobacter carboxidus is a soil Gram-negative alphaproteobacterium belonging to the physiological group of carboxydobacteria which aerobically oxidize CO to CO_2_. Here, we report the draft genome sequence of the *A. carboxidus* DSM 1086 type strain and the identification of both form I and form II CO dehydrogenase systems in this strain.

## ANNOUNCEMENT

Aminobacter carboxidus DSM 1086^T^ (basonym Carbophilus carboxidus), formerly known as Achromobacter carboxydus (1) or Alcaligenes carboxydus (2), is the type strain and the unique known member of the species (3, 4). It was isolated from soil near a stream in Moscow, Russia (1), and was assigned to the physiological group of carboxydobacteria due to its ability to grow aerobically on carbon monoxide (CO) as the sole carbon and energy source (1, 5). Carbon monoxide dehydrogenase (CODH) activity, which is responsible for the oxidation of CO to carbon dioxide (CO_2_), was formerly detected in *A. carboxidus* DSM 1086^T^ (6). Two CODH forms are known; form I specifically oxidizes CO, whereas form II is a putative CODH with a lower affinity for CO and still uncertain function (7, 8). Carbon dioxide produced by CO oxidation can be assimilated through the Calvin-Benson-Bassham cycle, although *A. carboxidus* is a facultative chemolithotroph able to utilize a wide variety of organic substrates for heterotrophic growth (5). Here, the genome sequence of *A. carboxidus* DSM 1086^T^ is reported with the aim of providing helpful insights into the genetic basis of CO oxidation in this monotypic strain.

*A. carboxidus* DSM 1086^T^ was obtained from DSMZ and aerobically grown at 30°C in Trypticase soy broth. DNA extraction was performed using a QIAamp DNA minikit (Qiagen). A genomic library of *A. carboxidus* was obtained with the TruSeq DNA PCR-free sample preparation kit (Illumina, Inc., San Diego, CA, USA). Genome sequencing was performed with a NextSeq 500 sequencing system (Illumina, UK) according to the manufacturer’s protocol, and library samples were loaded into a midoutput kit v2.5 (300 cycles) (Illumina, UK), producing 1,416,277 pairs of reads. Raw sequence reads were filtered and trimmed using the command-line fastq-mcf software (https://expressionanalysis.github.io/ea-utils/). Fastq files of Illumina paired-end reads (150 bp) were used as input in the MEGAnnotator pipeline for microbial genome assembly and annotation (9). This pipeline employed the SPAdes program v3.14.0 for *de novo* assembly of the genome sequence with the option “--careful” and a list of k-mer sizes of 21, 33, 55, 77, 99, and 127 (10). The genome quality was evaluated with the program CheckM (11), estimating a genome completeness of 99.3%. The contigs were then submitted to the National Center for Biotechnology Information (NCBI) for the prediction of protein-encoding open reading frames (ORFs) and tRNA and rRNA genes using the NCBI Prokaryotic Genome Annotation Pipeline (PGAP) (12). All tools were run with default parameters unless otherwise specified.

**FIG 1:**
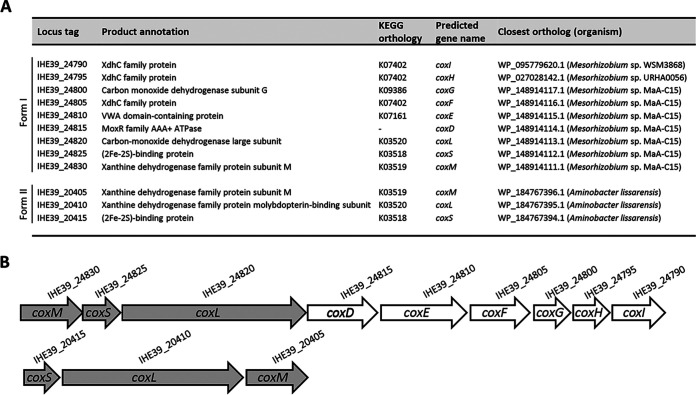
Forms I and II of the *cox* gene clusters in *A. carboxidus* DSM 1086^T^. (A) The putative *cox* genes encoding form I and form II of the heterotrimeric (αβχ)_2_ CODH enzyme complex (CoxL, CoxM, and CoxS subunits) of *A. carboxidus* DSM 1086^T^ with their GenBank annotation, gene name, and closest ortholog. KEGG Orthology numbers were assigned with the KEGG Automatic Annotation Server (KAAS) (13). (B) Physical map of the *A. carboxidus* DSM 1086^T^ genomic regions encompassing the form I (contig 11) and form II (contig 7) *cox* gene clusters. Form I is characterized by three structural genes, in the order *coxMSL* (gray), followed by six accessory genes (white), and by the presence of the AYXCSFR motif in the predicted CoxL active site. A different order of the three structural genes, *coxSLM* (gray), is characteristic of form II, and the CoxL active site contains the typical AYRGAGR motif.

The draft genome of *A. carboxidus* is 6,291,275 bp long. It was assembled into 31 contigs with an *N*_50_ value of 458,931 bp, an average coverage of 65×, and a mean GC content of 62.96%. Genome annotation identified 6,023 ORFs, 49 tRNA genes, and 3 rRNA genes. Two gene clusters predicted to encode both forms of heterotrimeric CODH were identified (Fig. 1). Form I showed the specific AYXCSFR signature in CoxL and six accessory genes (*coxDEFGHI*) flanking the *coxMSL* structural genes, whereas form II showed the typical *coxSLM* structural gene arrangement and the specific AYRGAGR signature in CoxL.

### Data availability.

This whole-genome shotgun project has been deposited at DDBJ/ENA/GenBank under accession number JACZEP000000000. The version described in this paper is JACZEP000000000.1. The raw sequencing reads are available at the Sequence Read Archive under accession number SRR12759717 and are associated with BioProject number PRJNA666410.

## References

[1] Zavarzin GA, Nozhevnikova AN 1977 Aerobic carboxydobacteria. Microb Ecol 3:305–326. doi:10.1007/BF02010738. 24233667

[2] Cypionka H, Meyer O 1983 The cytochrome composition of carboxydotrophic bacteria. Arch Microbiol 135:293–298. doi:10.1007/BF00413484.

[3] Meyer O, Stackebrandt E, Auling G 1993 Reclassification of ubiquinone Q-10 containing carboxidotrophic bacteria: transfer of “[*Pseudomonas*] *carboxydovorans*” OM5T to *Oligotropha*, gen. nov., as *Oligotropha carboxidovorans*, comb. nov., transfer of “[*Alcaligenes*] *carboxydus*” DSM 1086T to *Carbophilus*, gen. nov., as *Carbophilus carboxidus*, comb. nov., transfer of “[*Pseudomonas*] *compransoris*” DSM 1231T to *Zavarzinia*, gen. nov., as *Zavarzinia compransoris*, comb. nov., and amended descriptions of the new genera. Syst Appl Microbiol 16:390–395. doi:10.1016/S0723-2020(11)80271-7.

[4] Oren A, Garrity GM 2020 List of new names and new combinations previously effectively, but not validly, published. Int J Syst Evol Microbiol 70:4043–4049. doi:10.1099/ijsem.0.004244. 32731908

[5] Meyer O, Schlegel HG 1983 Biology of aerobic carbon monoxide oxidizing bacteria. Annu Rev Microbiol 37:277–310. doi:10.1146/annurev.mi.37.100183.001425. 6416144

[6] Kraut M, Hugendieck I, Herwig S, Meyer O 1989 Homology and distribution of CO dehydrogenase structural genes in carboxydotrophic bacteria. Arch Microbiol 152:335–341. doi:10.1007/BF00425170. 2818128

[7] King GM 2003 Molecular and culture-based analyses of aerobic carbon monoxide oxidizer diversity. Appl Environ Microbiol 69:7257–7265. doi:10.1128/aem.69.12.7257-7265.2003. 14660374 PMC309980

[8] King GM, Weber CF 2007 Distribution, diversity and ecology of aerobic CO-oxidizing bacteria. Nat Rev Microbiol 5:107–118. doi:10.1038/nrmicro1595. 17224920

[9] Lugli GA, Milani C, Mancabelli L, van Sinderen D, Ventura M 2016 MEGAnnotator: a user-friendly pipeline for microbial genomes assembly and annotation. FEMS Microbiol Lett 363:fnw049. doi:10.1093/femsle/fnw049. 26936607

[10] Bankevich A, Nurk S, Antipov D, Gurevich AA, Dvorkin M, Kulikov AS, Lesin VM, Nikolenko SI, Pham S, Prjibelski AD, Pyshkin AV, Sirotkin AV, Vyahhi N, Tesler G, Alekseyev MA, Pevzner PA 2012 SPAdes: a new genome assembly algorithm and its applications to single-cell sequencing. J Comput Biol 19:455–477. doi:10.1089/cmb.2012.0021. 22506599 PMC3342519

[11] Parks DH, Imelfort M, Skennerton CT, Hugenholtz P, Tyson GW 2015 CheckM: assessing the quality of microbial genomes recovered from isolates, single cells, and metagenomes. Genome Res 25:1043–1055. doi:10.1101/gr.186072.114. 25977477 PMC4484387

[12] Tatusova T, DiCuccio M, Badretdin A, Chetvernin V, Nawrocki EP, Zaslavsky L, Lomsadze A, Pruitt KD, Borodovsky M, Ostell J 2016 NCBI Prokaryotic Genome Annotation Pipeline. Nucleic Acids Res 44:6614–6624. doi:10.1093/nar/gkw569. 27342282 PMC5001611

[13] Moriya Y, Itoh M, Okuda S, Yoshizawa A, Kanehisa M 2007 KAAS: an automatic genome annotation and pathway reconstruction server. Nucleic Acids Res 35:W182–W185. doi:10.1093/nar/gkm321. 17526522 PMC1933193

